# The Effect of Probiotics on Symptoms, Gut Microbiota and Inflammatory Markers in Infantile Colic: A Systematic Review, Meta-Analysis and Meta-Regression of Randomized Controlled Trials

**DOI:** 10.3390/jcm9040999

**Published:** 2020-04-02

**Authors:** Karolina Skonieczna-Żydecka, Katarzyna Janda, Mariusz Kaczmarczyk, Wojciech Marlicz, Igor Łoniewski, Beata Łoniewska

**Affiliations:** 1 Department of Human Nutrition and Metabolomics, Pomeranian Medical University in Szczecin, 71-460 Szczecin, Poland; karzyd@pum.edu.pl (K.S.-Ż.); katarzyna.janda@pum.edu.pl (K.J.); sanprobi@sanprobi.pl (I.Ł.); 2 Department of Clinical and Molecular Biochemistry, Pomeranian Medical University in Szczecin, 70-111 Szczecin, Poland; mariush@pum.edu.pl; 3 Department of Gastroenterology, Pomeranian Medical University in Szczecin, 71-252 Szczecin, Poland; marlicz@hotmail.com; 4 Department of Neonatal Diseases, Pomeranian Medical University in Szczecin, 70-111 Szczecin, Poland

**Keywords:** infantile colic, probiotics, gut microbiota

## Abstract

Immaturity in digestive-tract motor function and altered intestinal microbiome may play roles in pathogenesis of infantile colic. We assessed the impact of probiotic therapy on crying duration day, in newborns experiencing colic attacks. The PubMed, Embase, Cinnahl, Web of Science databases, and a clinical trials registry (ClinicalTrials.gov) were searched from inception until 12/02/2020. Random-effects meta-analyses were used to derive standardized mean differences/differences in means and risk ratios. We included 16 studies, which involved 1319 newborns aged up to 6 months. *Lactobacillus reuteri* strain DSM17938 was administered predominantly (*n* = 10). Probiotic intervention reduced the duration of crying (standardized mean difference = −2.012, 95% confidence interval: −2.763 to −1.261, z = −5.25, *p* < 0.0001). The probability of at least a 50% reduction in crying duration was at least 1.98 times higher in the intervention group than in controls (Z = 4.80, *p* < 0.0001). The effects of the intervention were not significantly affected by the risk of bias assessment, percentage of breastfed infants, and duration of the study. In 11 studies, data concerning gut microbiota composition and function and/or immunological markers were given. Probiotics significantly shortened the crying duration, but a causal relationship between the modulatory effect of probiotics on microbiota and the immune system has not been confirmed.

## 1. Introduction

Infantile colic is a functional disorder of the gastrointestinal tract (point G4 in category G of the Rome IV Classification of Functional Gastrointestinal Disorders: Disorders of the Gut-Brain Interaction) [1,2,3]. Its apparent prevalence varies according to which diagnostic criteria are used and ranges from 2% to 73% [4]. The criteria for infantile colic were revised in the Rome IV classification and now, for clinical purposes, must include all of the following: (1) An infant who is less than 5 months of age when the symptoms start and finish; (2) Recurrent and prolonged periods of crying, fussing (see definition in Table S1), or irritability reported by caregivers that occur without obvious cause and which cannot be prevented or resolved by caregivers; (3) No evidence of failure to thrive, fever, or illness with clinical grounds (for details, see Table S1) [5].

Colicky ailments usually disappear in the first half-year of life, but before this happens, symptoms can bother parents, greatly resulting in repeated reporting to doctors [6,7,8]. The aetiology of intestinal colic in newborns is still poorly understood. It is presumed that the disorder results from a complex reaction between a newborn and the environment. Colic may also manifest as a result of immaturity of the structure and function of the gastrointestinal tract (GI) [9,10]. Both psychosociological factors, such as anxious parents, and gastrointestinal factors, such as allergy to cows’ milk or gastroesophageal reflux (GER), also play pathophysiological roles [11,12,13].

One of the most recent and intensively analysed theories assumes that newborn colic is partly due to alterations of gut microbiota. There is a body of evidence that links a colicky phenotype [14] with skewed microbiota composition (e.g., lower diversity) [15,16,17,18], excess gas production, and altered GI motility. Moreover, colicky newborns (CN) have been reported with alterations in bacterial abundance linked to gut inflammation and elevated levels of calprotectin and antimicrobial proteins released from intestinal neutrophils [15,16,17,19,20]. Of note, transplantation of CN’s faeces into mice resulted in visceral hypersensitivity to colorectal distention [21].

At least few interventions are known to alleviate excessive crying in colicky neonates, but with no evidence-based recommendations [22]. Historically, antispasmodic drugs, e.g., dicyclomine was found to counteract colicky behavior compared to placebo [23,24,25] but severe adverse effects, including coma [26], excluded the possibility to use the drugs in infants. As demonstrated in previous years [4,27,28], anti-foaming agents (simethicone), enzyme-based (lactase), anticholinergic-antimuscarinic-antispasmodic (cimetropium bromide, dicycloverine), opioids and antimuscarinic molecules (trimebutine), along with proton pump inhibitors (PPIs) showed some efficacy—as previously reported for simethicone [29,30]—but confirmed potentially dangerous adverse effects for dicyclomine, cimetropium [31] and PPIs [32].

Several previous studies [19,33,34,35,36], carried out to find evidence of the efficacy of various probiotic strains in relieving the symptoms of infantile colic, have produced conflicting results. Overall, meta-analyses have reported that microbial probiotics, predominantly including *Lactobacillus reuteri* strains, have significantly shortened crying duration in CN [37,38,39]. However, none of the conducted studies analysed mechanisms potentially associated with probiotic intervention, including gut microbiome analysis, immunological parameters, and intestinal barrier function. Additionally, new original research data have recently been published, which were not included in previous systematic reviews and meta-analyses.

The main aim of the current study was to prepare an updated systematic review and meta-analysis to evidence the effectiveness of probiotics in the treatment of colic, in order to confirm the hypothesis that probiotics are superior to placebo and result in shorter crying duration in CN. Additionally, in present metaanalysis, we have included studies looking for potential mechanisms of probiotics activity in CN.

Moreover, meta-regression was also carried out regarding age and percentages of breastfed newborns and the quality of included studies, in order to explain contributing factors that may influence probiotic efficacy.

## 2. Methods

### 2.1. Search Strategy and Inclusion Criteria

Two independent authors (KSZ, KJ) searched PubMed/Embase/CINAHL/Web of Science databases and a clinical trials registry (www.clinicaltrials.gov) from inception until 12/02/2020 for randomized controlled trials (RCTs), which compared the effects of probiotics/synbiotics (hereafter referred to together as “probiotics”) and placebos on reduction in crying duration in newborns manifesting colic attacks. The following search string was used in PubMed/Cinnahl/Web of Science: (“Probiotics”[MeSH] OR lactobacillus OR bifidobacterium OR saccharomyces OR streptococcus OR enterococcus OR bacillus) AND (“Colic”[MeSH] OR crying OR fussing OR irritable OR irritating). In Embase, the searched terms were: (‘infant’/exp OR ‘colic’/exp OR ‘abdominal colic’ OR ‘colic’ OR ‘colicky pain’ OR ‘gastrointestinal colic’) AND (‘probiotic agent’/exp OR ‘probiotic’ OR ‘probiotic agent’ OR ‘probiotics’) AND (‘placebo’/exp OR ‘placebo’) AND (‘crying’/exp OR ‘crying’ OR ‘weeping’ OR ‘fussiness’/exp). In the clinical trials registry, we used term “infantile colic” for condition and “probiotic” for other terms (https://clinicaltrials.gov/ct2/results?cond=infantile+colic&term=probiotic&cntry=&state=&city=&dist=). The electronic search was followed by a manual screen of relevant reviews. Inclusion criteria were: (i) full-text randomized controlled trial conducted with full-term CN; (ii) treatment with probiotic/synbiotic; (iii) randomization of probiotic vs. placebo; (iv) available meta-analyzable change score/endpoint data concerning crying duration (reduction in crying) during the day; and (v) English, German, or Polish text.

Data from studies containing more than two arms were collated separately for interventions. Additionally, if authors reported results for bottle-fed and breast-fed newborns separately, this representation was followed.

Exclusion criteria: (i) studies that randomized patients on more than one adjunctive intervention (e.g., probiotic plus proton pump inhibitor, probiotic incorporated into hydrolyzed casein formula); (ii) studies that did not conform to inclusion criteria.

### 2.2. Data Abstraction and Outcomes

We used the standard data extraction sheet according to our previous studies [40,41,42]. The following data from each study included were abstracted: study design, patient and treatment characteristics. For evaluation of the risk of bias (ROB) [43] we reported the number of low risk of bias assessments. Co-primary outcomes were crying duration per day and numbers of responders (defined as newborns who reduced daily crying by at least 50% compared to baseline) versus non-responders. These were assessed independently by at least two authors (K.S.-Z., K.J., I.Ł.), with one author (I.Ł.) acting as a dispute referee, in accordance with the Preferred Reporting Items for Systematic Reviews and Meta-Analyses (PRISMA). In the case of missing data, study authors were contacted via email. Data from figures was extracted by means of WebPlotDigitizer software (https://automeris.io/WebPlotDigitizer/).

### 2.3. Data Synthesis and Statistical Analyses

Using meta-analysis software (Comprehensive Meta-Analysis V3, Biostat, New Jersey, USA; www.meta-analysis.com), a random-effects [44] meta-analysis of outcomes, for which ≥3 studies contributed data, was conducted. Heterogeneity by means using chi-square tests of homogeneity was evaluated. Analyses were two-tailed and alpha was equal to 0.05.

For continuous outcomes, we analysed the pooled standardized mean difference (SMD) and difference in means (DM) in endpoint scores using observed cases (OC) data. For nominal outcomes the pooled risk ratio (RR), using OC data, was calculated. Meta-regression analyses with continuous (percentage of breastfed babies, study duration, number of low ROB assessments) covariates were conducted using random-effects models. The extent of asymmetries in funnel plots was detected using Egger’s tests.

## 3. Results

### 3.1. Search Results

**Figure 1:**
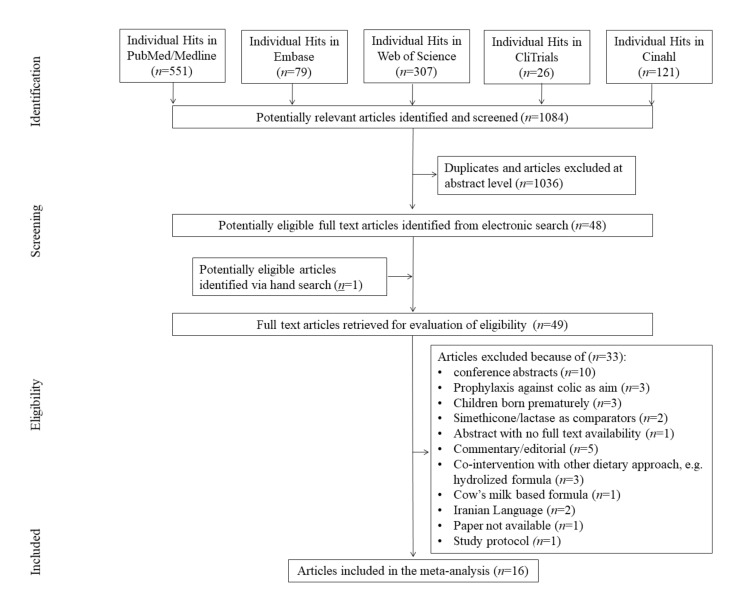
PRISMA flow diagram.

During the initial search, 1084 hits were found, of which 1036 were excluded at abstract level or as duplicates. The full-text review stage yielded 49 reviews. A total of 33 articles were excluded as they did not match inclusion criteria. We excluded all conference abstracts (*n* = 10). There were three studies (*n* = 3) conducted with premature newborns, which were excluded. Other reasons for exclusion were: studies aimed at preventing the incidence of colic (*n* = 4); co-intervention with other dietary approaches (*n* = 3); non-placebo comparators (*n* = 3); abstract with lack of full text availability (*n* = 1); commentary/editorials (*n* = 5), cows’ milk based formula (*n* = 1), Iranian Language (*n* = 2), study protocol (*n* = 1). We were unable to find one study’s full text (*n* = 1). In conclusion 16 studies were included in the final meta-analysis (see Figure 1, flow chart).

### 3.2. Study, Patient, and Treatment Characteristics

Ultimately 16 studies were included [19,33,34,35,45,46,47,48,49,50,51,52,53,54,55,56], the majority of which (*n* = 7) were conducted in Italy [19,33,45,46,49,50,54]. Intervention sponsorship was not specified for seven studies [19,45,47,48,50,51,53] and two trials [46,52] were financed by governmental institutions. There were 14 double-blinded studies [19,34,35,45,46,47,48,49,50,51,52,54,55,56], one single-blinded [53] and in one review, the blinding was not specified [33]. The duration of probiotic therapy was: median = 28 days, interquartile range (IQR) = 8 days, range = 14 to 90 days. The primary probiotic strain utilized in the included studies was *Lactobacillus reuteri* Deutsche Sammlung von Mikroorganismen (German Collection of Microorganisms; DSM) strain 17938 (*n* = 9) [19,33,34,35,47,49,50,52,53,56]. Other probiotic strains were: *Bifidobacterium breve* BR03 (DSM16604) and B632 (DSM24706) (*n* = 1) [45], *L. paracasei* DSM24733, *L. plantarum* DSM24730, *L. acidophilus* DSM24735, *L. delbrueckii* subsp. *bulgaricus* DSM24734, *B. longum* DSM24736, *B. breve* DSM24732, *B. longum* ssp. *infantis* DSMv24737 and *Streptococcus thermophilus* DSM24731 (*n* = 1) [46], *L. rhamnosus* GG (American Type Culture Collection: ATCC53103), *L. rhamnosus* LC705 (DSM7061), *B. longum* ssp. *infantis* Bbi99 (DSM13692; given as *B. breve* in [48]), and *Propionibacterium freudenreichii* ssp. *shermanii* JS (DSM7067; *n* = 1) [48]. A mix of *L. rhamnosus* 19070-2 and *L. reuteri* 12246 [55], and *B. animalis* subsp. lactis BB-12^®^, DSM 15954 were used in two studies, respectively [54]. In one study (*n* = 1), with a mixture of bacteria, there were no specific strain names given (*L. casei*, *L. rhamnosus*, *Streptococcus thermophilus*, *B. breve*, *L. acidophilus*, *B. longum* ssp. *infantis*, *L. bulgaricus,* and fructooligosaccharides) [51].

In total, 1319 newborns were randomized and 1215 analyzed, with a maximum of 172 newborns per study subjected to randomization and a mean ± SD of 82.44 ± 51.61 per study with a similar mean number for analysis (75.94 ± 54.06). The numbers of males and females were similar (males = 52.73%). In a majority of studies (*n* = 13) [19,33,34,35,46,47,48,49,50,51,53,55,56], infantile colic was diagnosed based on Wessel’s or modified-Wessel’s criteria. In one study, the Rome IV criteria were utilized [45], in one trial Rome III criteria [54] were used, and in one trial parental diaries were used for colic diagnoses [52]. As mentioned in the discussion, the inclusion criteria were only seemingly homogeneous (Table 1) and heterogeneity is discussed.

In 10 studies, newborns were breast-fed exclusively [33,46,47,48,49,50,51,52,54,55] and in other studies the percentage of naturally-fed newborns ranged from 59.28% to 87.2%, with the remaining newborns partly fed naturally and partly bottle-fed. Overall, probiotic intervention seemed to be well-tolerated as authors declared no adverse events regarding the intervention. In only one study [49], one newborn from a probiotic arm dropped out due to rhinitis, which was probably not related to the intervention. More study characteristics are shown in Table 1 and Table S2.

### 3.3. Risk of Bias (ROB)

The mean number of low risk-of-bias assessments in all studies included in the meta-analysis was 5.19 (median = 5) [19,34,35,46,47,48,49,50,51,52,53,54,55,56]. There were six studies with the highest number, i.e., seven low ROB assessments [34,46,47,51,52,54]. Details of ROB evaluation are given in Table S3.

### 3.4. Effects on Crying Duration and Response to Probiotic Intervention

**Figure 2:**
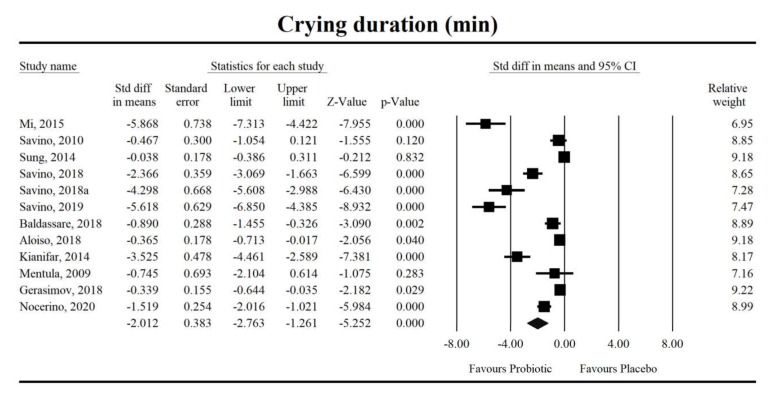
An effect size (random model), standardized mean difference, for crying duration in newborns taking probiotics vs. placebos (controls). Q = 227.3, df(Q) = 11, *p* < 0.001, I-squared = 95.2.

Using random-effects weights, the standardized mean difference for crying duration was −2.012 with a 95% confidence interval of −2.763 to −1.261 (z = −5.25, *p* < 0.0001; Figure 2). In case of difference in means, it was equal to −56.61 with a 95% confidence interval of −84.026 to −29.39 (z = −4.067; *p* < 0.0001). No covariates were associated with study-level effects of probiotics on crying duration for both effect sizes, respectively (SMD - ROB: coefficient = 0.15, standard error (SE) = 0.19, Z = 0.79, *p* = 0.43; breastfeeding percentage: coefficient = −0.03, SE = 0.03, Z = −1.20, *p* = 0.23; or duration of probiotic intervention: coefficient = 0.01, SE = 0.02, Z = 0.76, *p* = 0.45; DM - ROB: coefficient = 3.31, standard error (SE) = 7.21, Z = 0.46, *p* = 0.64; breastfeeding percentage: coefficient = −0.87, SE = 1.00, Z = −0.87, *p* = 0.39; or duration of probiotic intervention: coefficient = 0.65, SE = 0.71, Z = 2.05, *p* = 0.36).

**Figure 3:**
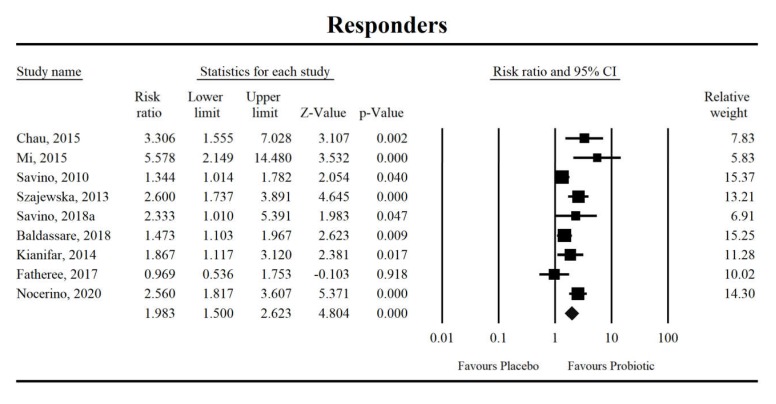
An effect size (random model), risk ratio, for the overall effects of probiotics with regard to a 50% reduction in crying duration. Q = 25.7, df (Q) = 8, *p* = 0.001, I-squared = 68.9.

Using random-effects analysis, the risk ratio for the eight studies was 1.98 with a 95% confidence interval of 1.5 to 2.62. The probability of at least a 50% reduction in crying duration was at least 1.81 times higher in the intervention group than in the control group (z = 4.804, *p* < 0.0001; Figure 3). A meta-regression using a random-effects model revealed no covariates regarding this study effect, respectively (ROB: coefficient = −0.18, SE = 0.21, Z = −0.84, *p* = 0.40; breastfeeding percentage: coefficient = −0.04, SE = 0.02, Z = −1.79, *p* = 0.07; or duration of probiotic intervention: coefficient = −0.03, SE = 0.02, Z = −1.35, *p* = 0.18).

**Figure 4:**
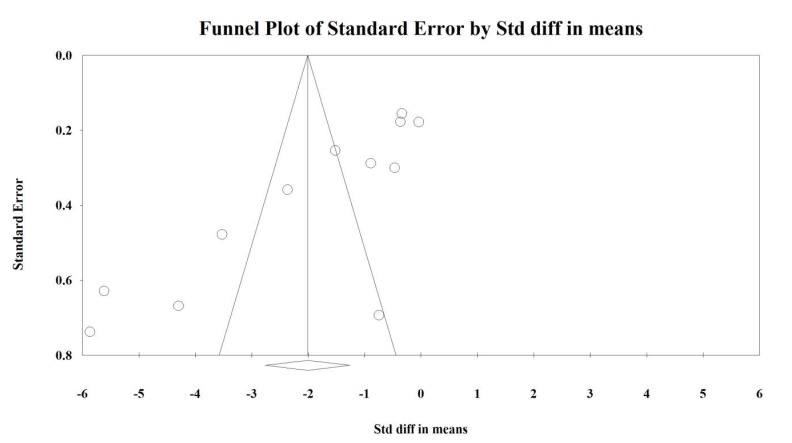
Funnel plot for crying time (SMD) in present meta-analysis.

**Figure 5:**
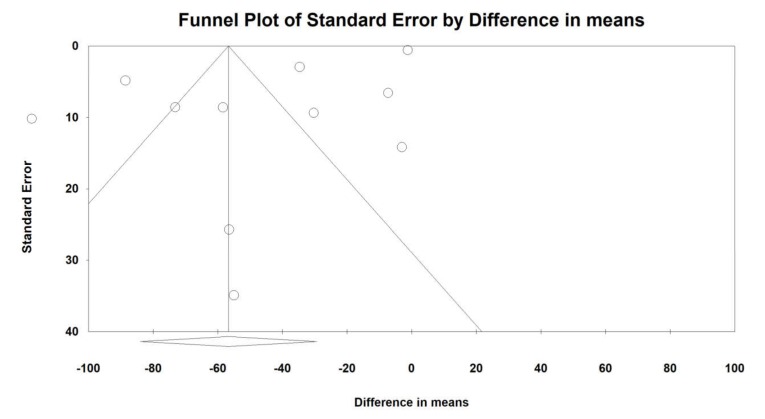
Funnel plot for crying time (DM) in present meta-analysis.

An Egger’s test suggested a publication bias regarding the net effect of probiotics on crying duration (Egger’s test - SMD: *p* = 0.0021; - DM: *p* = 0.007; Figure 4 and Figure 5) and there was no evidence of bias in the estimation of the response to intervention (Egger’s test *p* = 0.50; Figure S1).

### 3.5. Microbiota and Immunological Parameters

We found 11 trials [19,33,35,45,46,48,49,50,52,54,56], in which data concerning gut microbiota composition and function and/or immunological markers were given. These were data on faecal microbiota composition (*n* = 9), metabolomic analyses (*n* = 4) as well as faecal (calprotectin (*n* = 4)) and various blood immune (*n* = 5) markers. Table 2 presents major results on these parameters. Overall, aside from particular genera abundance [19,33,35,45,46,48,49,50,52,54,56], basic microbiota metrics, as alpha diversity [35,52,54], were reported. Only in three studies, microbiota by means of NGS technique was evaluated [35,52,54]. Other trials utilized a culture-dependent technique and qPCR. As one of the most important antimicrobial (and also a marker of gut inflammation and permeability [57,58] is calprotectin, we extracted raw data on this protein levels were possible (*n* = 5 studies). We found that the level of calprotectin decreased by probiotic treatment (*n* = 2 studies), but the data were presented in a not-metaanalyzable way (median, IQR) predominantly. These data are presented in Supplementary Table S4. We also analysed the association between clinical outcome, microbiota changes, and anti-inflammatory effects caused by probiotics administration (Supplementary Table S5). In three studies, clinical outcome was associated with microbial changes and in four studies with anti-inflammatory markers alterations. In three studies, changes in microbiota were observed, despite lack of clinical efficacy of probiotic treatment (in one study, crying time was even positively correlated with *L. reuteri* colonisation density). In two studies, statistical analysis of microbiota was not performed and in one study, the result of a metabolomic study was inconsistent (changes were also observed in the placebo group).

## 4. Discussion

This meta-analysis of 16 clinical trials and 1319 newborns exclusively investigated the impact of solely-probiotic interventions to reduce excessive crying in newborns manifesting symptoms of infantile colic, with no limitations on the age of study participants, and also gives a report on microbiota-related parameters affected by probiotics. Studies in which lactase, simethicone/herbal preparations or hydrolyzed formulas were utilized as probiotic comparators were not analyzed, as these interventions have their own evidence-based efficacy [59,60,61,62,63,64]. We also did not evaluate trials conducted with newborns who were not delivered at term, as premature newborns have been found to present immaturity in their digestive motility and interstitial Cajal cells [65] and are thus more prone to manifest behavioral perturbations [66]. By these means, we tried to diminish differences between populations of newborns in the articles studied which, as mentioned earlier, might have contributed to the uncertainty in probiotic efficacy found among previous reviews [67].

The results of the present meta-analysis indicated that probiotics significantly reduced the duration of crying per day when compared to placebos—as evaluated in a random–effects model. We were also able to show that the percentages of treatment responders were higher in the probiotic group. Overall, our primary study results are similar to what has been found in previous meta-analyses. Schreck Bird et al. [39] analyzed the efficacy of two *Lactobacillus reuteri* strains versus placebo or simethicone in newborns delivered at term and predominantly breast-fed. These authors found that a ~50% reduction in crying duration was >2 times more likely in newborns receiving probiotics. The authors, however, did not meta-analyze raw data concerning crying duration. Recently a similar study was published by Sung et al. [37], who evaluated *L*. *reuteri* DSM17938 as an agent that might relieve symptoms of infantile colic. The authors showed that after three weeks of intervention, the crying/fussing time decreased by almost half an hour. The incidence ratio to experienced relief was almost two times higher in the intervention group at different study points but significant in only breast-fed newborns. In the same year, a meta-analysis was conducted by Dryl and Szajewska [68] with seven RCTs and a cohort of 471 colicky participants, and found that crying reduction was significantly more probable in newborns taking probiotics and the number needed to treat was 5 (95% CI: 4–8). On average, newborns receiving probiotics cried about 50 minutes shorter when compared to newborns taking placebos. *Lactobacillus reuteri* DSM17938 efficacy, however, seemed to be present exclusively in breast-fed newborns. The most recent study published in the Cochrane database [38] evaluated the efficacy of probiotics with colic prevention in newborns aged up to 1 month in comparison to placebo, but the authors were not able to prove an association. On the other hand, a subgroup meta-analysis of six RCTs and a cohort of 707 newborns with age of up to 1 month showed that the mean crying duration per day decreased by 32.6 minutes (44.3 minutes in studies utilizing *Lactobacillus reuteri* strains) in newborns receiving probiotics. Overall, the authors declared that no clear evidence of probiotic efficacy exists.

In our review, we found that probiotic effects were significant for both newborns exclusively breast-fed and newborns predominantly fed naturally; however, a meta-regression concerning the percentage of breastfed infants suggested that the efficacy of probiotics might be greater in infants fed with formulas, although the coefficient was extremely low (Coefficient = −0.04; *p* = 0.07). In one study [45], included in our review, the authors reported that they had analyzed bottle- and breast-fed newborns separately but reported outcomes only in naturally-fed newborns. In another study [58], the crying duration was portrayed in such a way that meta-analysis adjustments were not possible concerning method of feeding. In the above-mentioned meta-analysis by Sung et al. [37], the authors found that the effects of probiotics were not significant in exclusively artificially-fed newborns, which cannot be confirmed by our review because we did not analyze exclusively bottle-fed newborns. As breastfeeding may potentially influence the efficacy of probiotics in IC, we can therefore make a presumption that the probiotic phenomenon might be associated with gut microbiota, which differ in naturally-fed newborns in comparison to artificially-fed newborns. Indeed, mother’s milk contains its own microbiome [69,70] and also most importantly indigestible human milk oligosaccharides [71], which favourably affect the gut ecosystem [72]. Additionally, there are data that state that artificial feeding promotes the development of coliforms in CN, and that this microbial print has not been demonstrated in breast-fed newborns with colic or newborns free of colic attacks [73]. On the other hand, if breast milk microbiota are affected by intrapartum antibiotics, in some cases, the beneficial effect of breast milk on the gut microbiome may be unfavourably altered [74]. Importantly, most authors did not report variables that may shape gut microbiota and intestinal permeability in newborns, e.g., mode of delivery, weight gain during pregnancy, antibiotic therapy during pregnancy and labor [75], which all may affect colicky behaviour. In addition, a body of evidence exists that links infantile colic with caffeine consumption [76] and smoking [77,78], as well as consumption of allergenic products [79]. Apart from in a few trials, we did not find much information regarding mother’s health and lifestyle in the studies we included. In particular, in one study, the parents were advised to avoid cow’s milk [49], while another excluded mothers taking antibiotics or those with allergy to cow’s milk [50]. In a study by Sung et al. [35], the authors demonstrated that (by a small percentage difference) hypoallergenic formulas were more commonly used in the placebo group, as well as a dairy-free diet by mothers of breast-fed newborns. These might be factors that potentially biased the apparent overall effects of probiotics against prolonged colicky crying in breast-fed newborns.

Last but not least, we abstracted data related to influence of probiotics administration on gut microbiota and immunological markers. The most frequently studied variables were: (i) the effects of probiotic administration on the composition of the microbiota; (ii) colonization with probiotics, and (iii) the effect of reducing colonization with bacteria considered to be associated with colicky phenotype (e.g., *E. coli* [54]). Among immunological markers, mainly calprotectin and markers of Treg lymphocyte function were assessed. Based on the results obtained, no definite association can be found between the use of probiotics, microbiota changes, modulation of the immune system, and either presence or lack of clinical effects (Table 2 and Tables S4–S5). Of note, the results cannot be subjected to meta-analysis due to very diverse methods used to analyze the microbiota. Therefore, the results are difficult to compare. In addition, it is important to state that changes of microbiota are very dynamic in the first months and years of human life [80]. These observations are backed by studies assessing immunological markers such as calprotectin in this group of patients [81,82]. In addition, these markers are dependent on maternal-fetal factors, such as antibiotic therapy or BMI changes during pregnancy, mode of delivery, and feeding patterns [75]. For this reason, in order to fully assess the causal relationship between the microbiota and the function of the immune system with relation to symptoms of infantile colic, a multifactorial analysis should be performed, which was not performed in the works described in this systematic review. In only two studies, the correlation between microbiota changes and the effectiveness of probiotics was demonstrated [54,56]; however, the results were contradictory. In addition, the results of metabolomic studies did not contribute to elucidation of the mechanism of action of probiotics studied. Therefore, it cannot be determined whether the effect of probiotics in infantile colic is related to their effect on microbiota or the immune system. The relationship observed in some studies is rather based on association not causation. We conclude that mechanistic studies should be an important point in analysis of probiotics efficacy in CN.

One study from the present meta-analysis diagnosed colic using Rome IV criteria [45] and the other Rome III criteria [54]. The rest utilised Wessel’s or modified Wessel’s criteria, and in one trial, a parental diary on crying duration was used. However, the reports on gut microbiota included in the above-mentioned papers all seem to be in line with the newest Rome IV criteria released in 2016 (Table S1). These criteria state that functional gastrointestinal disorders are the consequence of altered gut-brain interactions, an umbrella term for motility disorders, visceral hypersensitivity, immune dysfunction, microbiota alterations, and central nervous system alterations [2]. In a study based on Rome IV criteria [45], it was demonstrated that a probiotic strain, *Lactobacillus reuteri* DSM17938, may have had a positive impact on crying duration in CN, underlining the hypothesis that gut-brain axis dysfunction may play a role in a colic phenotype. As has been documented that a major role of gut microbiota is to shape the structure and function of the gut and nervous system, we hope that the role of microbiota in this pathogenesis of CN will finally be elucidated, providing probiotics as a documented dietary option.

Several limitations of this meta-analysis require consideration. These include (i) a relatively small number of double-blinded studies comparing probiotic intervention to placebo; (ii) heterogeneous study inclusion criteria, particularly the age of newborns enrolled, and the type of strain and duration of probiotic intervention; (iii) the meta-regression analyses were only exploratory, as they were based on studies that may differ in newborn and treatment characteristics (delivery mode, antibiotic therapy as well as species/dosage/trial duration) from the overall sample in relevant ways.

In fact, all of these limitations may serve as confounding factors for probiotic efficacy; thus, to clearly show the efficacy of such microbial intervention, it is essential to conduct studies with restricted inclusion criteria, homogenous feeding, and probiotics to describe in great detail the impact of probiotics on colicky behavior. Additionally, these factors may have resulted in the asymmetrical funnel plot regarding crying duration. As elegantly reviewed by Sterne et al. [83], there are several possible explanations for such asymmetry, including reporting bias, poor methodological quality, true heterogeneity, as well as artifacts and chance. We carefully evaluated the risk of bias of each study and overall consider the quality of the included trials as high. We presume that intervention protocols differed significantly and this variable might remain as a so-called small study effect. Lastly, during meta-analysis, we did not use intent-to-treat data, which were not reported by authors but are preferred in clinical trials if the objective is pragmatic. We are also aware of potential bias during the review process, as we might have missed some studies not clearly aimed at reducing crying duration in CN, but which might describe such outcomes.

## 5. Conclusions

Despite the limitations, this is a comprehensive meta-analysis evaluating solely-probiotic interventions compared to placebo, which showed that such dietary interventions are beneficial for CN and may counteract excessive crying. The mechanism of the action of probiotics in CN is still unknown. Based on the current analysis, we cannot assume that the action of probiotics is mediated either through modulation of microbiota or immune function. Relationships observed in some trials are rather based on associations and not causations. Therefore, further studies based on strict inclusion criteria are needed to clarify the role of probiotics in infantile colic and their impact on the gastrointestinal tract (e.g., intestinal barrier); and to compare the effectiveness of individual probiotic strains and doses, time of administration, and corresponding therapeutic effects.

## Figures and Tables

**Table 1:** Study characteristics.

Study (Country)	Study Description					Sample Description			Intervention	
	Design, Blinding	ROB^	n Randomized/ Analyzed	Duration (Days)	Study Focus	Age (Mean ± SD, Days)	Male (%)	Breast-fed (%)	Probiotic Name	Probiotic Dose
Aloiso et al., 2018 [45] (Italy)	DB	4	158/155	90	efficacy against functional gastrointestinal disorders	10.5 ± 2.15	52.26	83.87	*B. breve* BR03 (DSM 16604) and *B. breve* B632 (DSM 24706)	drops containing 10^8^ CFU of each strain
Baldassare et al., 2018 [46] (Italy)	DB	7	62/53	21	efficacy in infantile colic	38.75 ± 1.72	58.49	100	*L. paracasei* DSM 24733, *L. plantarum* DSM 24730, *L. acidophilus* DSM 24735, and *L. delbrueckii* subsp. *bulgaricus* DSM 24734), three strains of bifidobacteria (*B. longum* DSM 24736, *B. breve* DSM 24732, and *B. longum* ssp. *infantis* DSM 24737), and one strain of *Streptococcus thermophilus* DSM 24731	5 × 10^9^ CFU/10 drops
Chau et al., 2015 [47] (Canada)	DB	7	55/52	21	efficacy in infantile colic	39.73 ± 0.32	48.08	100	*L. reuteri* DSM 17938	10^8^ CFU in 5 drops/day
Fatheree et al., 2017 [52] (USA)	DB	7	20/16	42	efficacy against crying, fussing, inflammatory, immune, and microbiome variables	Probio: 57 (39. 72); PBO: 40 (34. 51) ††	75	100	*L. reuteri* DSM 17938	10^8^/day
Gerasimov et al., 2018 [55] (Ukraine)	DB	5	172/168	28	efficacy against crying, fussing	44.5 ± 15	50	100	*L. rhamnosus* 19070-2, *L. reuteri* 12246	250 × 10^6^ CFU + 3.33 mg FOS+200IU vitamin D3
Kianifar et al., 2014 [51] (Australia)	DB	7	50/45	30	reduction of newborns crying over time	42.17 ± 17.38	48.89	100	*L. casei*, *L. rhamnosus*, *Streptococcus thermophilus*, *B. breve*, *L. acidophilus*, *B. longum* ssp. *infantis*, *L. bulgaricus* and FOS	10^9^ CFU/sachet
Mentula et al., 2008 [48] (Finland)	DB	0	18.wrz	14	reduction of newborns crying over time and effect on gut microbiota	21 ± nd	33.33	100	L. rhamnosus GG, L. rhamnosus LC705, B. longum ssp. infantis Bbi99, and Propionibacterium freudenreichii ssp. shermanii JS	*L. rhamnosus* GG:5 × 10^9^ CFU; *L. rhamnosus* LC705: 5 × 10^9^ CFU; *B. breve* Bbi99: 2 × 10^8^ CFU; *P. freudenreichii* ssp. *shermanii* JS: 2 × 10^9^ CFU
Mi et al., 2015 [53] (China)	SB	5	42/39	28	efficacy in infantile colic	29.16 ± 15.59	56.41	87.18	*L. reuteri* DSM 17938	10^8^ CFU
Nation et al. 2017 [56] (Australia)	DB	5	167/167	28	The relationship between *L. reuteri* colonisation and crying time, microbial and inflammatory parameters ***	50.18 ± 19.06	50.89	59.28	*L. reuteri* DSM 17938	0.2 × 10^8^ CFU/day
Nocerino et al., 2020 [54] (Italy)	DB	7	80/78	28	efficacy in infantile colic	32.95 ± 5.15	55.12	100	*Bifidobacterium animalis* subsp. *lactis* BB-12^®^, DSM 15954	10^9^ CFU/day
Savino et al. 2010 [49] (Italy)	DB	6	50/46	21	efficacy in infantile colic and its relationship to the gut microbiota	PBO: 28.5 (21) Probio: 32.5 (21) †	63.04	100	*L. reuteri* DSM 17 938	10^8^ CFU
Savino et al., 2018 [19] (Italy)	DB	5	87/60	30	reduction of newborns crying and modifying the RORg/FOXP3 expression, gut microbiota and faecal calprotectin	47.06 ± 23.4	43.33	83.33	*L. reuteri* DSM 17938	10^8^ CFU/ drop; 5 drops/day
Savino et al., 2018a [50] (Italy)	DB	5	59/30	28	influence on Treg and TLR expression (TLR 2 and TLR4)	26.4 ± 12.36	40	100	*L. reuteri* DSM 17938	0.2 × 10^8^ CFU/drop; 5 drops
Savino et al., 2019 [33] (Italy)	nd	1	50/50	28	influence on CC-chemokine receptor 7 (CCR7) and interleukin 10 (IL-10)	<50; PBO: 28.5 (21) Probio: 32.5 (21) †	58	100	*L. reuteri* DSM 17938	10^8^ CFU/ drop; 5 drops/day
Sung et al., 2014 [35] (Multicenter)	DB	5	167/167	28	reduction of newborns crying and fussing	50.18 ± 19.06	50.90	59.28	*L reuteri* DSM 17938	0.2 × 10^8^ CFU/drop; 5 drops/day
Szajewska et al., 2013 [34] (Poland)	DB	7	82/80	21	efficacy in infantile colic	36.2 ± 12.25	60	86.25	*L. reuteri* DSM 17938	10^8^ CFU in 5 drops

†—median (IQR); ††—median (Q1-Q3); ^—number of low risk of bias (ROB) assessments; FOS—fructooligosaccharides; CFU—colony forming units; DB—double-blinded; Probio—probiotic; PBO—placebo; SB—single-blinded; nd—not determined, ***—partly same cohort as Sung et al., 2014

**Table 2:** Microbiological (compositional and functional) and inflammatory biomarkers affected by probiotics.

Reference	Microbiota (Faeces)/Immunological Markers *	Method
Aloisio et al., 2018 [45]	Whole cohort: ↑ probiotic *B. breve* counts. Bottle fed newborns: ↓ potentially pathogenic *B. fragilis* group members. ↑ *Bifidobacterium* spp. and ↓ *Clostridium* spp over time with no treatment effect, no differences in *E. coli* abundance over time.	qPCR
Baldassare et al., 2018 [46]	No differences in total bacteria counts and *Bifodobacterium* spp. over time and treatment. A statistical insignificant tendency toward elevated *Lactobacillus* spp. with probiotic supplementation. ↑ faecal propylene glicol in probiotic group and ↑ alanine, 2-hydroxyisovalerate and 2-oxoisocaproate in placebo group.	qPCR/H-NMR
Fatheree et al., 2017 [52]	No differences in faecal alpha-diversity (Shannon, Chao1, or Simpson diversity indices) over time and by treatment ↓ of dominant faecal gram (-) bacteria, i.e., *Klebsiella*, *Proteus*, and *Veillonella* ↓ faecal calprotectin over time but not by the Tx blood: ↓ IL-2; ↓ population of Tregs: CD4^+^Foxp3^+^CD25^+^ within CD4^+^Foxp3^+^, and CD4^+^Foxp3^+^HELIOS^+^ within CD4^+^Foxp3^+^ (thymus-derived) with colic resolution in probiotic treated group (no statistical analysis was done)	NGS/flow cytometry/ELISA
Mentula et al., 2009 [48]	↑ of total counts of anaerobic bacteria, bifidobacteria, *L. rhamnosus* GG, enterococci. Faecal fermentation parameters (SCFA, CFA) were measured but no statistical analysis was performed.	culture-dependent technique/GC
*** Nation et al., 2017 [56]	Crying time reduction regardless *L. reuteri* colonisation No differences in *E. coli* colonization rates or densities and microbial diversity regarding *L. reuteri* colonization status. *E. coli* density negatively correlated with microbial diversity *L. reuteri* concentration positively correlated with crying time No difference in faecal calprotectin levels regarding probiotic colonization status	qPCR/T-RFLP/ELISA
Nocerino et al., 2020 [54]	No difference in microbiota composition and alpha-diversity index by Tx ↑ *Bifidobacterium* spp. only in the responder infants treated with BB-12 *Bifidobacterium* abundance was correlated with the reduction of crying time ↑ Proteobacteria in the placebo group ↑ butyrate levels in responders Blood: ↑ HBD-2, LL-37, sIgA levels and ↓n faecal calprotectin level in responders	high-throughput sequencing of 16S rRNA, ELISA, indirect enzyme immunoassays
Savino et al., 2010 [57]	↑ in *Lactobacillus* spp. (including probiotic *L. reuteri*) by Tx ↓ in faecal *E. coli* and ammonia	culture-dependent techniques, enzymatic colorimetric test
Savino et al., 2018 [19]	↑ of *Lactobacillus* spp. by T ↓ feacal calprotectin Blood: ↑ of FOXP3 concentration thus decreased RoRg/FOXP3 mRNA ratio	qPCR, ELISA
Savino et al., 2018a [50]	- Blood: ↑ mRNA expression of TREGs, FOXP3	real time PCR/Qpcr
Savino et al., 2019 [33]	-↑ expression of CC-chemokine receptor 7	qPCR
Sung et al., 2014 [58]	No differences between in faecal microbial diversity, and *E. coli* load by Tx ↓ feacal calprotectin in responders from probiotic and placebo group	16SrDNA amplification (T-RFLP) ELISA, qPCR

IL-2 = interleukin 2; CD4+Foxp3+CD25+ =; CD4+Foxp3+HELIOS+ within CD4+Foxp3+ =; Helios-positive (thymus-derived) Tregs =; SCFA = short chain fatty acids; CFA = cellular fatty acids; HBD-2 = human β-defensin 2; LL-37 = cathelecidin; sIgA = secretory IgA; FOXP3 = forkhead box P3; RORγ = retinoid-related orphan receptor-γ; mRNA = messenger RNA; Th17 = T helper cell 17; Treg = regulatory T cell; qPCR = quantitative polymerase chain reaction; Tx – treatment; T-RFLP = terminal restriction fragment length polymorphism; NGS = next generation sequencing. rDNA = ribosomal DNA; ELISA = enzyme-linked immunosorbent assay; *—when not specified faecal biomarkers are listed; ***—partly same cohort as Sung et al., 2014

## Supplementary Materials

The following are available online at https://www.mdpi.com/2077-0383/9/4/999/s1, Table S1: Diagnostic criteria for newborn colic according to Rome IV, Rome II, and Wessel’s criteria; Table S2: All-cause and adverse-effects-cause discontinuation within the trials; Table S3: Risk of bias assessment; Table S4: Faecal calprotectin levels (µg/g) by probiotic treatment; Table S5: Summary of the clinical outcome and changes in microbial composition and metabolites as well as anti-inflammatory effects associated with probiotics administration. Figure S1: Funnel plot for responding rate in present meta-analysis.
