# Supplementary material for: The Effect of Probiotics on Symptoms, Gut Microbiota and Inflammatory Markers in Infantile Colic: A Systematic Review, Meta-Analysis and Meta-Regression of Randomized Controlled Trials
Source: J Clin Med. 2020 Apr 2;9(4):999. doi: 10.3390/jcm9040999 (PMC7231167; doi:10.3390/jcm9040999)
Supplement: Supplementary file 1 [file jcm-09-00999-s001.pdf]

Table S1. Diagnostic criteria for newborn colic according to Rome IV, Rome II and Wessel's criteria [1,2]**.**

Rome IV criteria	Rome III criteria	Wessel's criteria
For clinical purposes, must include all of the following:<br>1. A newborn who is less than 5 months of age when the symptoms start and stop.<br>2. Recurrent and prolonged periods of crying, fussing (see below), or irritability reported by caregivers that occur without obvious cause and cannot be prevented or resolved by caregivers.<br>3. No evidence of newborn failure to thrive, fever, or illness "Fussing" refers to intermittent distressed vocalization and has been defined as "[behavior] that is not quite crying but not awake and content either." Newborns often fluctuate between crying and fussing, so that the two symptoms are difficult to distinguish in practice.<br><br>For clinical research, a diagnosis of newborn colic must meet the preceding diagnostic criteria and also include both of the following:<br>1. Caregiver reports newborn has cried or fussed for 3 or more hours/day during 3 or more days within 7 days in a telephone or face-to-face screening interview with a researcher or clinician.<br>2. Total 24-hour crying plus fussing in a selected newborn is confirmed to be 3 hours or more when measured by at least one prospectively kept 24-hour behavior diary.	Diagnostic criteria must include all of the following in newborns from birth to 4 months of age:<br>1. Paroxysms of irritability, fussing, or crying that start and stop without obvious cause.<br>2. Episodes lasting 3 or more hours/day and occurring at least 3 days/week for at least 1 week.<br>3. No failure to thrive.	Intense, unexplained fussing and crying lasting more than 3 hours per day for more than 3 days per week, for more than 3 weeks.

Table S2. All-cause and adverse-effects-cause discontinuation within the trials.

Reference	Discontinuation reason: All cause				Discontinuation reason: Adverse Events			
	Probiotic, group cases	Probiotic, n	Placebo, group cases	Placebo, n	Probiotic, group cases	Probiotic, n	Placebo, group cases	Placebo, n
Aloiso et al.., 2018 [3]	3*	82	3*	73	0	82	0	73
Baldassare et al., 2018 [4]	5	31	4	31	0	27	0	26
Chau et al., 2015 [5]	3	27	0	28	0	24	0	28
Fatheree et al., 2017 [6]	1	13	2	7	0	13	0	7
Gerasimov et al., 2018[7]	2	86	2	86	0	86	0	86
Kianifar et al., 2014 [8]	1	26	4	24	0	25	0	20
Mentula et al.., 2008 [9]	9*	5	9*	4	nd	nd	nd	nd
Mi et al., 2015 [10]	1	21	2	21	0	21	0	21
Nation et al., 2017[11]	18	85	22	82	0	85	0	82
Nocerino et al., 2020[12]	0	40	2	40	0	40	0	40
Savino et al., 2010 [13]	0	25	4	25	1#	25	4##	25
Savino et al., 2018 [14]	0	32	6	28	0	32	0	28

Savino et al., 2018 [15]	0	18	4	16	0	18	4[###]	16
Savino et al., 2019 [16]	0	25	0	25	0	25	0	25
Sung et al., 2014 [17]	18	85	22	82	0	85	0	82
Szajewska et al., 2013 [18]	0	40	0	40	0	42	0	40

#: rhinitis; ##: eczema (n=1), fever (n=1), otalgy (n=1), gastroesophageal reflux (n=1); ###: fever (n=4); *the number of all subjects that dropped out (group was not specified); nd: not determined.

Table S3. Risk of bias assessment.

Study reference.	Random sequence generation (selection bias).	Allocation concealment (selection bias).	Blinding of participants and personnel (performance bias).	Blinding of outcome assessment (detection bias).	Incomplete outcome data addressed (attrition bias) (Short-term outcomes (2-6 weeks)).	Selective reporting (reporting bias).	Other bias.
Aloiso et al., 2018 [3]	L	L	L	L	H	?	?
Baldassare et al., 2018 [4]	L	L	L	L	L	L	L
Chau et al., 2015 [5]	L	L	L	L	L	L	L
Fatheree et al., 2017 [6]	L	L	L	L	L	L	L
Gerasimov et al., 2018[7]	L	L	L	?	L	L	?
Kianifar et al.., 2014 [8]	L	L	L	L	L	L	L
Mentula et al.., 2008 [9]	?	?	?	?	?	?	H
Mi et al., 2015 [10]	L	?	L	?	L	L	L
Nation et al., 2017[11]	L	L	L	L	?	?	L

Nocerino et al., 2020[12]	L	L	L	L	L	L	L
Savino et al., 2010 [13]	L	L	L	L	L	?	L
Savino et al., 2018 [14]	L	L	L	L	H	H	L
Savino et al., 2018 [15]	L	L	L	L	?	?	L
Savino et al., 2019 [16]	H	H	H	H	L	?	?
Sung et al., 2014 [17]	L	L	L	L	?	?	L
Szajewska et al., 2013 [18]	L	L	L	L	L	L	L

L: low risk of bias; H: high risk of bias; ?: unclear risk of bias.

Table S4. Faecal calprotectin levels (μg/g) by probiotic treatment.

**Reference**	PROBIOTIC			PLACEBO			p value
	Median (IQR)		N	Median (IQR)		N	
	Baseline	Endpoint		Baseline	Endpoint		
Fatheree et al.,2017[6]	216 (134)	Day 42: 140 (78; 251)*<br>Day 92: 75 (48; 118)*	13	148 (110)	Day 42: 633 (102;172)*<br>Day 92: 94 (58; 150)*	7	0.19#<br>0.5^; 0.57^^
Nocerino et al., 2020 [12]	832.36 (590)	930.63 (784)	40	777.46 (590)	1023.12 (825)	38	<0.05##
Savino et al., 2018 [19]	541 (429.5)	165 (246.8)	22	361 (376.5)	182 (321.5)	32	<0.001###
Sung et al., 2014 [20]	nd	135 (185)	53	nd	114 (169)	49	0.63
Nation et al.2017[11]	CHILDREN COLONIZED WITH PROBIOTIC STRAIN		N	CHILDREN NOT COLONIZED WITH PROBIOTIC STRAIN		N	p value
	Baseline	Endpoint		Baseline	Endpoint		

nd	135.4 (479)	13	nd	114.8 (170.8)	45	0.37

IQR-interquartile range, *data are means (95% Confidence interval) # baseline probiotics vs. baseline placebo, ^ - day 42: endpoint probiotics vs. endpoint placebo; ^^ day 92: endpoint probiotics vs. endpoint placebo ##- probiotics baseline vs. probiotics endpoint; placebo baseline vs. placebo endpoint; probiotics endpoint vs. placebo endpoint; ### probiotics baseline vs. probiotics endpoint; nd – not determined.

Table S5. Summary of the clinical outcome and changes in microbial composition and metabolites as well as anti-inflammatory effects associated with probiotics administration.

Reference	Clinical outcome	Microbiota composition changes	Microbial metabolites changes	Anti-inflammatory activity	Comments
Aloiso et al., 2018 [3]	N	O	N	N	↑ abundance of probiotic bacteria and ↓abundance of potential pathogenic bacteria only in one subgroup, changes of some bacteria independently from the treatment effect
Baldassare et al., 2018 [4]	O	N	?	N	Results of metabolomic studies are inconsistent (changes of some markers in placebo group)
Fatheree et al., 2017 [6]	N	?	N	N	Only suggestion that probiotic treatment can shape Tregs.
Mentula et al., 2009 [9]	N	O	?	N	Faecal bacteria fermentation parameters (SCFA, CFA) were measured but no statistical analysis was performed.
Nation et al., 2017 [11]	N	Crying time was positively correlated with *L. reuteri* colonisation density	N	N	Different study model in comparison to other studies: the relationship between *L. reuteri* colonisation and crying time, microbial as well as inflammatory parameters were analysed
Nocerino et al., 2020[12]	O	O	O	O	*Bifidobacterium* abundance was correlated with the reduction of crying time.
Savino et al., 2010[21]	O	O	O	N	-
Savino et al., 2018[19]	O	O	N	O	-
Savino et al., 2018a[22]	O	N	N	O	-

Savino et al., 2019[23]	O	N	N	O	-
Sung et al., 2014[20]	N	N	N	N	After one month symptoms increased in probiotic group. Lower calprotectin level was observed in responders from probiotic and placebo group

N-not observed, O-observed, ? – results are not clear, Tregs - regulatory T cell, SCFA-short chain fatty acid, CFA-cellular fatty acids.

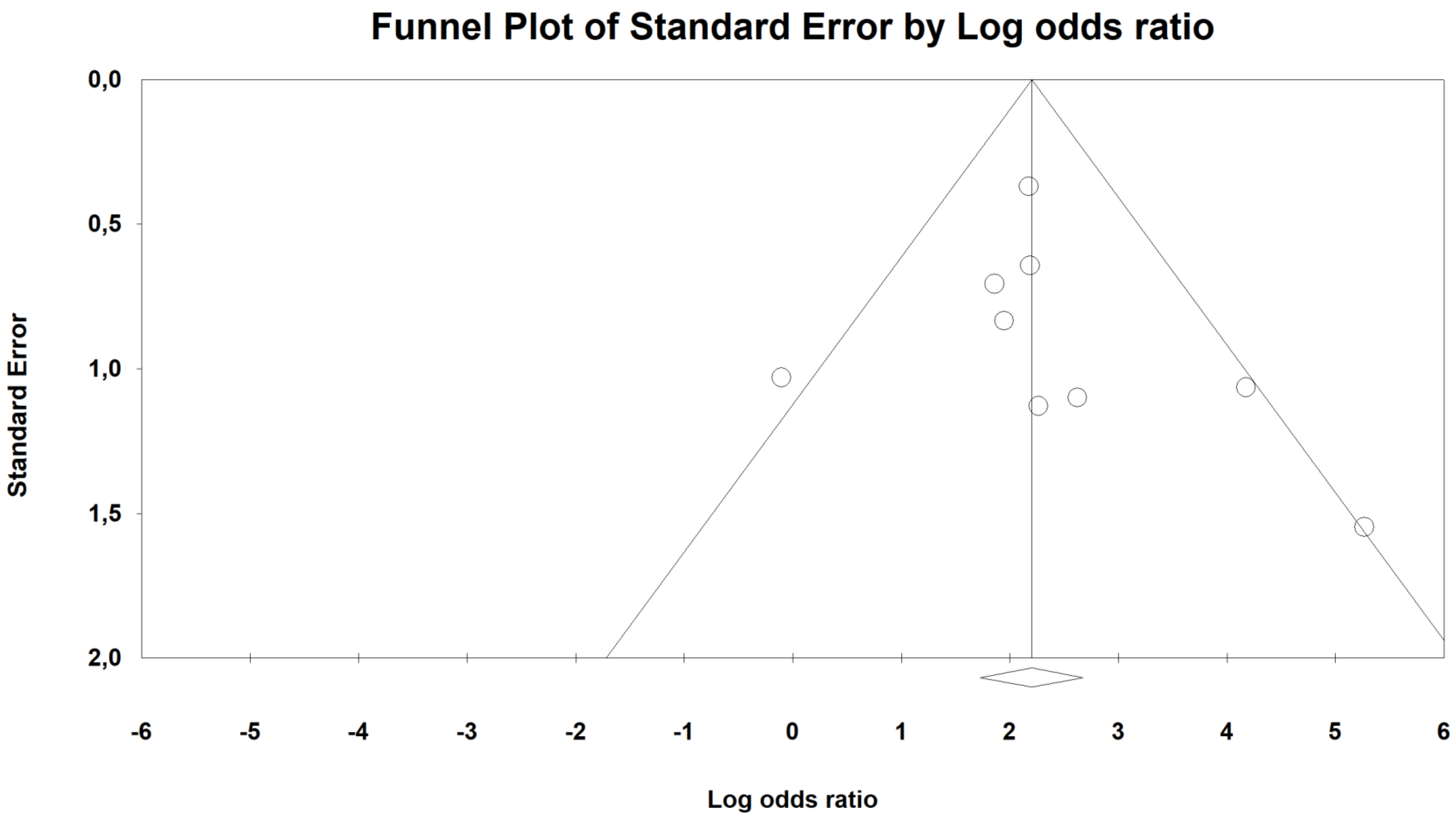

Figure S1. Funnel plot for responding rate in present meta-analysis.

Supplementary references.

1. Palsson, O.S.; Whitehead, W.E.; van Tilburg, M.A.L.; Chang, L.; Chey, W.; Crowell, M.D.; Keefer, L.; Lembo, A.J.; Parkman, H.P.; Rao, S.S.; et al. Rome IV Diagnostic Questionnaires and Tables for Investigators and Clinicians. *Gastroenterology* **2016**.
2. Wessel, M.A.; Cobb, J.C.; Jackson, E.B.; Harris, G.S.; Detwiler, A.C. Paroxysmal fussing in infancy, sometimes called colic. *Pediatrics* **1954**, *14*, 421–435.
3. Aloisio, I.; Prodam, F.; Giglione, E.; Bozzi Cionci, N.; Solito, A.; Bellone, S.; Baffoni, L.; Mogna, L.; Pane, M.; Bona, G.; et al. Three-Month Feeding Integration With Bifidobacterium Strains Prevents Gastrointestinal Symptoms in Healthy Newborns. *Front Nutr* **2018**, *5*, 39.
4. Baldassarre, M.E.; Di Mauro, A.; Tafuri, S.; Rizzo, V.; Gallone, M.S.; Mastromarino, P.; Capobianco, D.; Laghi, L.; Zhu, C.; Capozza, M.; et al. Effectiveness and Safety of a Probiotic-Mixture for the Treatment of Infantile Colic: A Double-Blind, Randomized, Placebo-Controlled Clinical Trial with Fecal Real-Time PCR and NMR-Based Metabolomics Analysis. *Nutrients* **2018**, *10*, 195.
5. Chau, K.; Lau, E.; Greenberg, S.; Jacobson, S.; Yazdani-Brojeni, P.; Verma, N.; Koren, G. Probiotics for infantile colic: a randomized, double-blind, placebo-controlled trial investigating Lactobacillus reuteri DSM 17938. *J. Pediatr.* **2015**, *166*, 74–78.
6. Fatheree, N.Y.; Liu, Y.; Taylor, C.M.; Hoang, T.K.; Cai, C.; Rahbar, M.H.; Hessabi, M.; Ferris, M.; McMurtry, V.; Wong, C.; et al. Lactobacillus reuteri for Infants with Colic: A Double-Blind, Placebo-Controlled, Randomized Clinical Trial. *J Pediatr* **2017**, *191*, 170-178.e2.
7. Gerasimov, S.; Gantzel, J.; Dementieva, N.; Schevchenko, O.; Tsitsura, O.; Guta, N.; Bobyk, V.; Kaprus, V. Role of Lactobacillus rhamnosus (FloraActive™) 19070-2 and Lactobacillus reuteri (FloraActive™) 12246 in Infant Colic: A Randomized Dietary Study. *Nutrients* **2018**, *10*, 1975.
8. Kianifar, H.; Ahanchian, H.; Grover, Z.; Jafari, S.; Noorbakhsh, Z.; Khakshour, A.; Sedaghat, M.; Kiani, M. Synbiotic in the management of infantile colic: A randomised controlled trial. *J Paediatr Child Health* **2014**, *50*, 801–805.
9. Mentula, S.; Tuure, T.; Koskenala, R.; Korpela, R.; Könönen, E. Microbial composition and fecal fermentation end products from colicky infants – a probiotic supplementation pilot. *Microb Ecol Health Dis* **2008**, *20*, 37–47.
10. Mi, G.-L.; Zhao, L.; Qiao, D.-D.; Kang, W.-Q.; Tang, M.-Q.; Xu, J.-K. Effectiveness of Lactobacillus reuteri in infantile colic and colicky induced maternal depression: a prospective single blind randomized trial. *Antonie Van Leeuwenhoek* **2015**, *107*, 1547–1553.
11. Nation, M.L.; Dunne, E.M.; Joseph, S.J.; Mensah, F.K.; Sung, V.; Satzke, C.; Tang, M.L.K. Impact of Lactobacillus reuteri colonization on gut microbiota, inflammation, and crying time in infant colic. *Sci Rep* **2017**, *7*, 15047.
12. Nocerino, R.; De Filippis, F.; Cecere, G.; Marino, A.; Micillo, M.; Di Scala, C.; de Caro, C.; Calignano, A.; Bruno, C.; Paparo, L.; et al. The therapeutic efficacy of Bifidobacterium animalis subsp. lactis BB-12® in infant colic: A randomised, double blind, placebo-controlled trial. *Aliment. Pharmacol. Ther.* **2020**, *51*, 110–120.
13. Savino, F.; Cordisco, L.; Tarasco, V.; Palumeri, E.; Calabrese, R.; Oggero, R.; Roos, S.; Matteuzzi, D. Lactobacillus reuteri DSM 17938 in Infantile Colic: A Randomized, Double-Blind, Placebo-Controlled Trial. *Pediatrics* **2010**, *126*, e526–e533.
14. Savino, F.; Garro, M.; Montanari, P.; Galliano, I.; Bergallo, M. Crying Time and RORγ/FOXP3 Expression in Lactobacillus reuteri DSM17938-Treated Infants with Colic: A Randomized Trial. *J. Pediatr.* **2018**, *192*, 171-177.e1.
15. Savino, F.; Galliano, I.; Garro, M.; Savino, A.; Daprà, V.; Montanari, P.; Bergallo, M. Regulatory T cells and Toll-like receptor 2 and 4 mRNA expression in infants with colic treated with Lactobacillus reuteri DSM17938. *Benef Microbes* **2018**, *9*, 917–925.
16. Savino, F.; Galliano, I.; Savino, A.; Daprà, V.; Montanari, P.; Calvi, C.; Bergallo, M. Lactobacillus reuteri DSM 17938 Probiotics May Increase CC-Chemokine Receptor 7 Expression in Infants Treated With for Colic. *Front Pediatr* **2019**, *7*, 292.
17. Sung, V.; Hiscock, H.; Tang, M.L.K.; Mensah, F.K.; Nation, M.L.; Satzke, C.; Heine, R.G.; Stock, A.; Barr, R.G.; Wake, M. Treating infant colic with the probiotic Lactobacillus reuteri: double blind, placebo controlled randomised trial. *BMJ* **2014**, *348*, g2107.
18. Szajewska, H.; Gyrczuk, E.; Horvath, A. Lactobacillus reuteri DSM 17938 for the Management of Infantile Colic in Breastfed Infants: A Randomized, Double-Blind, Placebo-Controlled Trial. *J Pediatr* **2013**, *162*, 257–262.
19. Savino, F.; Garro, M.; Montanari, P.; Galliano, I.; Bergallo, M. Crying Time and RORγ/FOXP3 Expression in Lactobacillus reuteri DSM17938-Treated Infants with Colic: A Randomized Trial. *J. Pediatr.* **2018**, *192*, 171-177.e1.
20. Sung, V.; Hiscock, H.; Tang, M.L.K.; Mensah, F.K.; Nation, M.L.; Satzke, C.; Heine, R.G.; Stock, A.; Barr, R.G.; Wake, M. Treating infant colic with the probiotic Lactobacillus reuteri: double blind, placebo controlled randomised trial. *BMJ* **2014**, *348*, g2107–g2107.

21. Savino, F.; Cordisco, L.; Tarasco, V.; Palumeri, E.; Calabrese, R.; Oggero, R.; Roos, S.; Matteuzzi, D. Lactobacillus reuteri DSM 17938 in infantile colic: a randomized, double-blind, placebo-controlled trial. *Pediatrics* **2010**, *126*, e526-533.
22. Savino, F.; Galliano, I.; Garro, M.; Savino, A.; Daprà, V.; Montanari, P.; Bergallo, M. Regulatory T cells and Toll-like receptor 2 and 4 mRNA expression in infants with colic treated with Lactobacillus reuteri DSM17938. *Beneficial Microbes* **2018**, *9*, 917–925.
23. Savino, F.; Galliano, I.; Savino, A.; Daprà, V.; Montanari, P.; Calvi, C.; Bergallo, M. Lactobacillus reuteri DSM 17938 Probiotics May Increase CC-Chemokine Receptor 7 Expression in Infants Treated With for Colic. *Front Pediatr* **2019**, *7*.
